# Arsenic trioxide reverses the chemoresistance in hepatocellular carcinoma: a targeted intervention of 14–3-3η/NF-κB feedback loop

**DOI:** 10.1186/s13046-018-1005-y

**Published:** 2018-12-20

**Authors:** Yongxin Qiu, Yi Dai, Chi Zhang, Ye Yang, Ming Jin, Wenqi Shan, Jian Shen, Ming Lu, Zhaoyang Tang, Liang Ju, Yuting Wang, Ruonan Jiao, Yunwei Xia, Guangming Huang, Lihua Yang, Yuan Li, Jianping Zhang, Vincent Kam Wai Wong, Zhihong Jiang

**Affiliations:** 1 The Second Affiliated Hospital of Nanjing Medical University, Nanjing, China; 2 Jiangsu Key Lab of Cancer Biomarkers, Prevention and Treatment, Jiangsu Collaborative Innovation Center For Cancer Personalized Medicine, School of Public Health, Nanjing Medical University, Nanjing, China; 3 State Key Laboratory of Quality Research in Chinese Medicine, Macau Institute for Applied Research in Medicine and Health, Macau University of Science and Technology, Macau, China

**Keywords:** Hepatocellular carcinoma, Arsenic trioxide, Multi-drug resistance, 14–3-3η, Nuclear factor kappa B

## Abstract

**Background:**

Multi-drug resistance (MDR) is one of the main obstacles for treatment of advanced/recurrent hepatocellular carcinoma (HCC). We have previously identified arsenic trioxide (ATO) as an effective metastasis/angiogenesis inhibitor in HCC. Here, we further found that MDR-HCC cells were more sensitive to ATO.

**Methods:**

The MDR-HCC cells were used as experimental models. Biological functions were investigated using cell transfection, polymerase chain reaction, western blot, southwestern blot, immunostaining, immunoprecipitation plus atomic fluorescence spectrometry, and so on.

**Results:**

The MDR-HCC cells underwent high oxidative stress condition, and employed adaptive mechanisms for them to survive; while ATO abolished such mechanisms via targeting the 14–3-3η/nuclear factor kappa B (NF-κB) feedback Loop. Briefly, in MDR cells, the increase of ROS activated NF-κB signaling, which transcriptionally activated 14–3-3η. Meanwhile, the activation of NF-κB can be constitutively maintained by 14–3-3η. As a NF-κB inhibitor, ATO transcriptionally inhibited the *14–3-3η* mRNA level. Meanwhile, ATO was also validated to directly bind to 14–3-3η, enhancing the degradation of 14–3-3η protein in an ubiquitination-dependent manner. Knockdown of 14–3-3η reduced the ATO-induced reversal extents of drug resistance in MDR cells.

**Conclusion:**

14–3-3η/NF-κB feedback loop plays an important role in maintaining the MDR phenotype in HCC. Moreover, via targeting such feedback loop, ATO could be considered as a potential molecular targeted agent for the treatment of HCC.

**Electronic supplementary material:**

The online version of this article (10.1186/s13046-018-1005-y) contains supplementary material, which is available to authorized users.

## Background

Hepatocellular carcinoma (HCC) is one of the most common malignant tumors and a leading cause of cancer-related mortality worldwide [1]. Surgical operation is the radical cure of HCC, nevertheless, most of the patients lose the surgical indications due to the advanced-stage of detection [2]. Currently, the transcatheter hepatic arterial chemoembolization (infusion of 5-fluorouracil, oxaliplatin, and/or doxorubicin) is the main non-operative methods for clinical treatment of advanced HCC [3]. However, the overall survival is not obviously ameliorated largely owing to the multiple-drug resistance (MDR) [4–6]. Therefore, unraveling the potential mechanisms, and identifying the novel therapeutic strategies to overcome MDR are urgently needed.

The 14–3-3 family (seven isoforms: β, γ, σ, ε, ζ, η, and τ) are the phosphine/threonine binding proteins of approximate 28 to 33 kDa acidic polypeptides [7]. By binding to the Ser-X-pSer or Ser-X-pThr (X represents an arbitrary amino acid) residue, they regulate multiple cellular functions, such as the regulation of cell cycle progression, the initiation and maintenance of DNA damage checkpoints, the prevention of apoptosis, and cytoskeletal dynamics [8]. Up to date, six members of 14–3-3 family proteins (β, γ, σ, ε, ζ, and η) have been identified to be involved in promoting the growth, angiogenesis, and metastasis of HCC [9]. Among them, two members (ζ and σ) of 14–3-3 proteins have also been identified to be associated with chemotherapy/molecular target drug resistances [10, 11]. In particular, our previous study revealed that, by forming a positive feed-back loop with extracellular signal-regulated kinase 1/2 (ERK1/2), 14–3-3η effectively promotes the growth, angiogenesis, and sorafenib resistance [12]. Therefore, in our present study, we continued to reveal the effects and underlying mechanisms of 14–3-3η on MDR in HCC, and further aimed to find out potential interventions.

## Methods

### Regents

Arsenic trioxide (ATO, As_2_O_3_, > 99.0% purity), 5-fluorouracil (5-Fu, C_4_H_3_FN_2_O_2_, > 99.0% purity), oxaliplatin (L-OHP, C_8_H_14_N_2_O_4_Pt, > 99.0% purity), and doxorubicin (DOX, C_27_H_29_NO_11_·HCl, > 98.0% purity) were purchased from Sigma-Aldrach (Shanghai, China). The BAY-117082 and MG-132 were purchased from Beyotime Co. Ltd. (Haimeng, China). The stattic, topotecan, and ML-385 were obtained from Med-Chem-Express (Shanghai, China). All other reagents used were of analytical grade or the highest grade available.

### Cell culture and construction of 5-Fu-resistant cells

HCC cell line, Bel-7402 was obtained from Institute of Biochemistry and Cell Biology, Chinese Academy of Science (Shanghai, China), and was STR identified by China Center for Type Culture Collection (Wuhan, China). Cells were maintained in a 37 °C humidified incubator with 5% CO_2_ in RPMI-1640 medium (Gibco, Grand Island, NY), supplemented with 10% fetal bovine serum (FBS), 100 U/ml penicillin, 100 μg/ml streptomycin (Gibco). To obtain 5-Fu-resistant cells (Bel/5-Fu), as described previously [13–15], the Bel-7402 cells were cultured in RPMI-1640 medium with 2 μM of 5-Fu for 5 days. After an exponential growth was again reached in these treated cells, RPMI-1640 medium with 20 μM of 5-Fu was added and cells was cultured for another period of time. Likewise, the cells were gradually exposed to stepwise increasing concentrations for a harsh selection. After a continuous induction of approximately 5 months, Bel/5-Fu cells were successfully established after a sustained growth and steady passage in the RPMI-1640 medium contained 200 μM of 5-Fu.

### Cell transfection

Scrambled and pcDNA-3.1-14-3-3η-Flag were synthesized by Generay Biotech (Shanghai, China); while 14–3-3η- and NF-κB/p65 siRNA were purchased from Santa Cruz Biotechnology (http://datasheets.scbt.com/sc-43581.pdf, and https://datasheets.scbt.com/sc-29410.pdf) [12, 16]. Cells were transiently transfected via lipofectamine 2000 reagent (Invitrogen, Carlsbad, USA), according to the manufacturer’s protocol (Note: plasmids used: 5 ng/ml. siRNAs used: 20 nM). After transfection, the cells were cultured in fresh medium supplemented with 10% FBS for another 24 h before being used for other experiments.

#### Calculation of the 50% inhibitory dose (IC_50_)

Cell viabilities were determined via using a CCK-8 kit [12]. The IC_50_s were calculated via a graph-pad 6.0 software (CA, USA). For each test, the inhibition ratio was determined via a three-parameter dose-response equation and calculated with a non- linear regression. A sigmoidal curve was generated and the IC_50_ value was acquired. The ordinate represented inhibition ratio, while the abscissa represented the log (concentrations). The calculation mode used was “log (inhibitor) *vs*. response (three parameters)”, and results exhibited represented “best-fit values” ± “std. errors”.

### Determination of the intracellular ROS

The ROS Assay Kit was purchased from Beyotime Co. Ltd. As we described previously, treated cells were incubated in triplicate with DCFH-DA at 37 °C for 30 min, then the fluorescent signal was observed using a fluorescence microscope (Olympus, Tokyo, Japan), and the DCFH fluorescence intensity was measured by a multi-well plate reader (Bio-Rad) at an excitation wavelength of 488 nm and an emission wavelength of 525 nm [17, 18].

### Quantitative real-time polymerase chain reaction (qRT-PCR)

The isolation of total RNA, the transcription of RNA to cDNA, and the performance of qRT-PCR with Applied Biosystems 7300HT machine were all according to our previous study [19]. The primers for 14–3-3η were F: 5′-cctgcctcttagccaaac-3′, and R: 5′-ctcctgcttcttcatcctg-3′. The β-actin was amplified to ensure cDNA integrity and to normalize expression. Fold changes in expression of each gene were calculated by a comparative threshold cycle (Ct) method using the formula 2^-(ΔΔCt)^.

### Western blot (WB) and southwestern blot (SB)

Extraction of total/nuclear proteins, measurement of their concentrations with BCA kit, and SDS-PAGE followed by transferring the protein to PVDF membranes were all according to our previous study [20]. The primary antibodies used were: 14–3-3η, p-IKKβ, p-IκBα, p-p65 (Cell Signaling Technology, MA, USA, dilutions, 1: 1000), Flag, and β-actin (Beyotime Co. Ltd., the dilutions, 1: 2000). The biotin-labeled probe (contained the promoter of 14–3-3η: 5′-ccctcggcgttgtccgcggc-3′) was synthesized by Zhuoli Biotechnology Co., Ltd. (Shanghai, China). The immune complexes were detected by enhanced chemiluminescence (Cell Signaling Technology) and densitometric analysed via an image-pro-plus 6.0 software (Media Cybernetics, Georgia, USA) [21].

### Immunostaining

After cells were fixed, permeablized, and blocked as described previously [12], they were incubated with rabbit-anti-p65 (Cell Signaling Technology, dilution, 1: 200) antibody at 4 °C overnight, followed by incubating for 1 h with FITC-conjugated anti-rabbit secondary antibody (Beyotime Co. Ltd.; dilutions, 1: 500). The nuclei were stained by adding 4′, 6-diamidino-2-phenylindole (DAPI, Beyotime Co. Ltd). Then, cells were observed and pictured under a Zeiss-700B laser scanning confocal microscope (Zeiss Co. Ltd., Oberkochen, Germany).

### Immunoprecipitation plus atomic fluorescence spectrometry (IP-AFS)

After Bel/5-Fu cells were pre-treated by 0 or 20 μM of MG-132, for 2 h, they were exposed to 10 μM of ATO for 6 h. Then, such cells were extracted for 30 min with immunoprecipitation lysis buffer (Beyotime Co. Ltd). After centrifugation of the preparations, 100 μg of total proteins were incubated with 14–3-3η antibody (dilution, 1: 100) at 4 °C overnight. Then the protein-antibody complexes were incubated with IgG sepharose beads (Beyotime Co. Ltd) at 4 °C for another 12 h. After then, the supernatants were removed (positive control) and the beads were washed for three times (residual supernatants, served as a negative control), boiled to remove protein from the beads and extracted by 5 M of HCl (experimental group). Then we added 5 M of KOH to the extraction to adjust the pH between 2.0–7.0.

For hydride generation atomic fluorescence spectrometry, we used the dual channel atomic fluorescence spectrometer with special arsenic high intensity hollow cathode lamp (Titan Instruments Co, Beijing, China). The instrument working parameters were: pre-reducing agent (3.5% HCl + 0.1% ascorbic acid+ 0.1% thiourea), reducing agent (1% KBH4+ 0.175% KOH), carrier gas (argon gas), PMT voltage (260 V), primary current (50–80 mA), atomizer height (8 mm), carrier gas flow rate (500 ml/m), shield gas flow rate (800 ml/m), sampling time (9 s), delay period (2 s), measurement mode (peak area), and determination method (standard curve, STD).

### Statistical analysis

Data were presented as the means ± SD. The differences were analyzed via using student’s t test, one-way analysis of variance followed by Dunnett’s t test, or two-way analysis of variance followed by Sidak’s multiple comparisons test via a graph-pad 6.0 software. The *p* values < 0.05 were considered statistically significant.

## Results

### Characterization of 5-Fu-resistant HCC cells

**Fig. 1:**
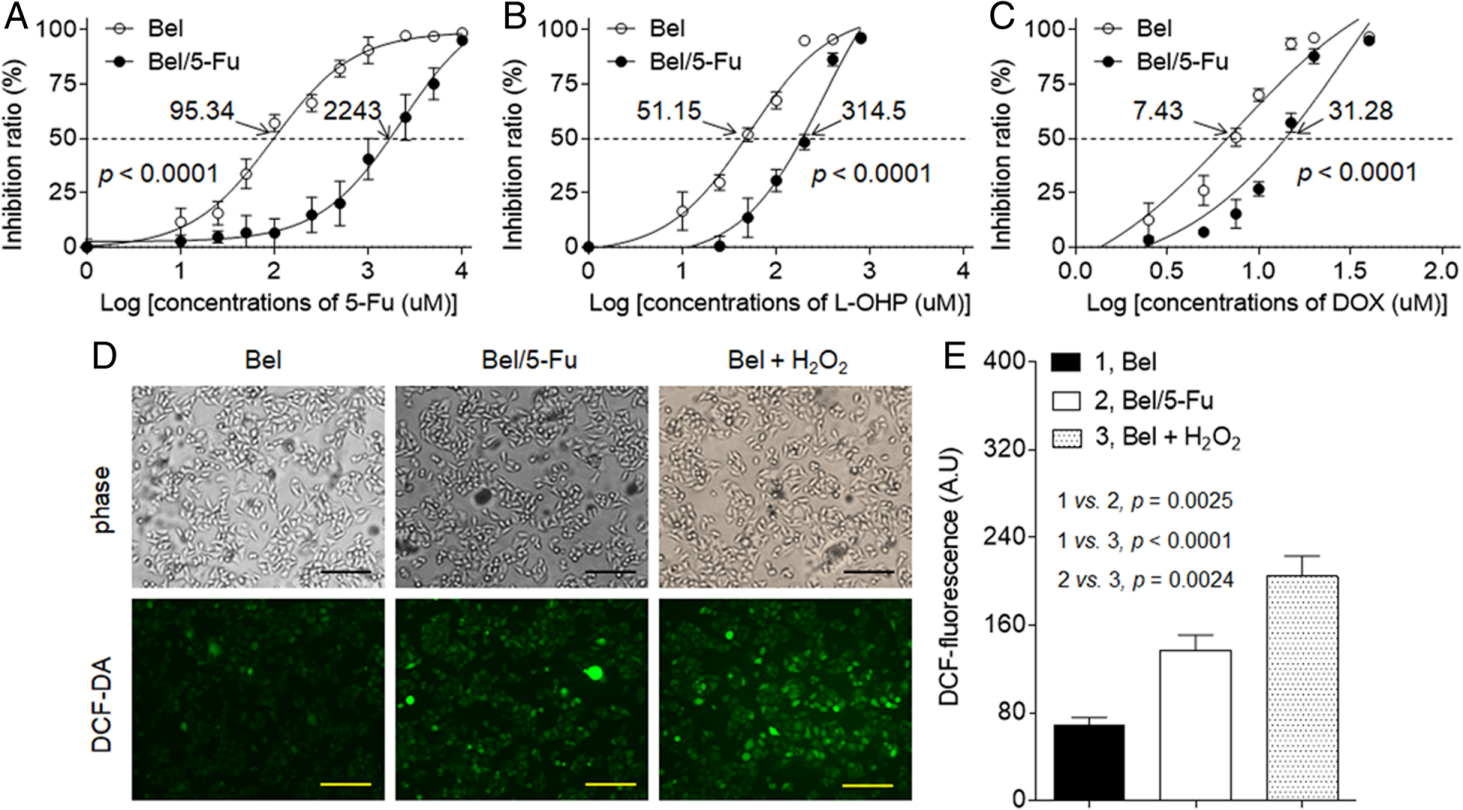
Characterization of 5-Fu-resistant HCC cells: **a** to **c** Bel-7402 or Bel/5-Fu cells were treated by different concentrations of 5-fluorouracil (0 to 10^4^ μM), oxaliplatin (0 to 10^3^ μM), or doxorubicin (0 to 10^2^ μM) for 24 h, respectively. The cell viability was determined in triplicate, and the IC_50s_ were calculated. **d** and **e** The intracellular ROS levels were determined in triplicate (the H_2_O_2_-treated Bel-7402 cells were used as a positive control; Bars = 250 μm)

Cell viability assays demonstrated that the 5-Fu-resistant Bel-7402 cells (Bel/5-Fu) were more resistant to chemotherapeutic drugs (5-fluorouracil, oxaliplatin, and doxorubicin) than its parental cells, Bel-7402. The IC_50s_ (μM) of these three drugs for Bel-7402 and Bel/5-Fu cells were: 5-fluorouracil (95.34 vs. 2243, Fig. 1a), oxaliplatin (51.15 vs. 314.5, Fig. 1b), and doxorubicin (7.43 vs. 31.28, Fig. 1c). Moreover, compared with Bel-7402 cells, increased expressions of MDR related genes, such as *MDR1* and *MRP1* were observed in Bel/5-Fu cells (Additional file 1: Figure S1). So, we confirmed that the Bel/5-Fu cells obtained the MDR phenotype. In addition, the intracellular ROS level (as determined by DCF-fluorescence) was elevated in Bel/5-Fu cells in comparison with its parental counterparts (Fig. 1d and e). Thus, we further suggested that Bel/5-Fu cells were exposed to relative higher oxidative stress condition, which further facilitated these cells to employ the adaptive mechanisms for survival in an inhospitable microenvironment.

### Effects of 14–3-3η on anti-oxidation and MDR in HCC cells

**Fig. 2:**
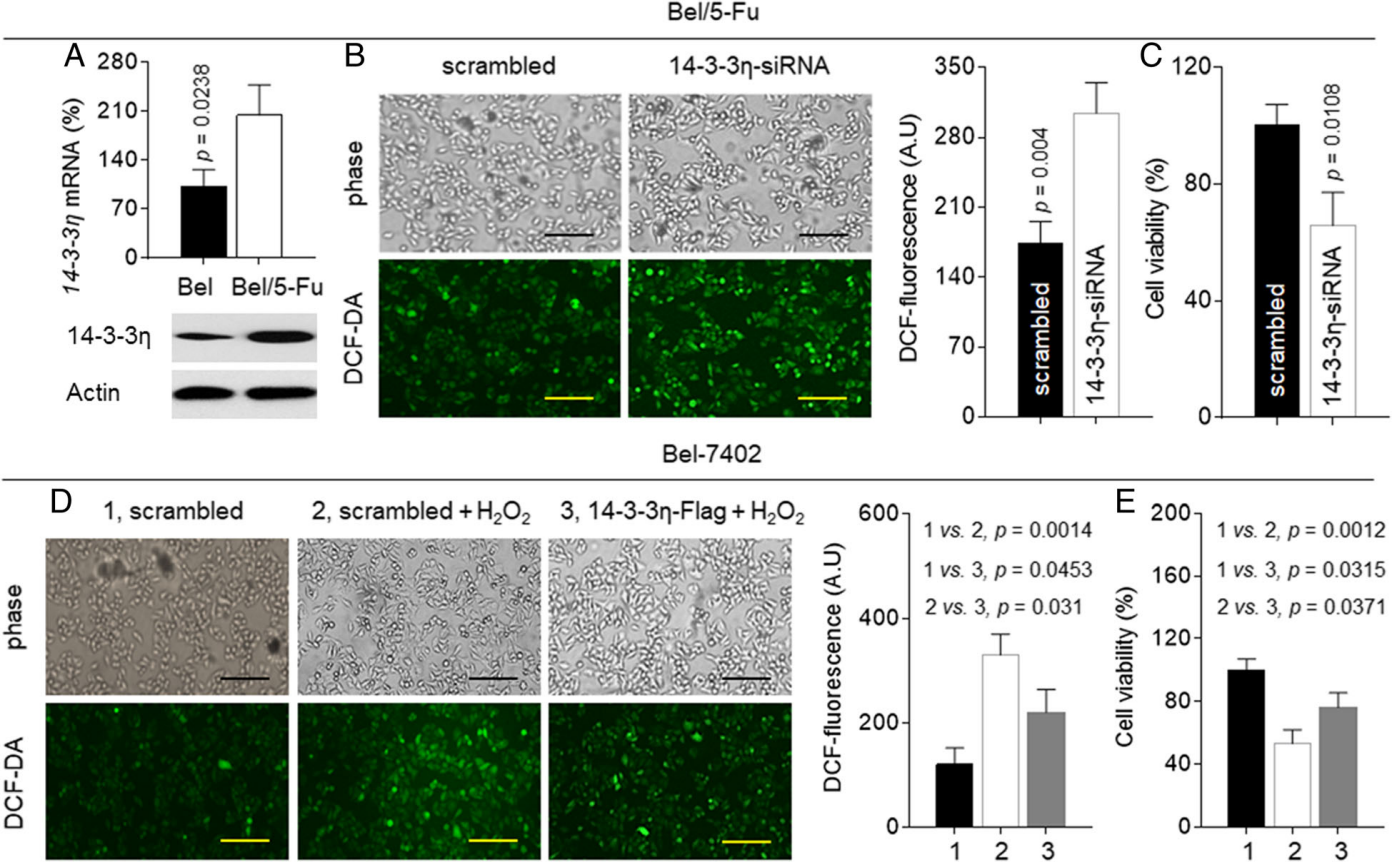
Effects of 14–3-3η on antioxidation in HCC cells: **a** qRT-PCT analyses in triplicate and Western blot analyses of the expressions of 14–3-3η mRNA (top) and protein (bottom). **b** and **c** Bel/5-Fu cells were transfected by scrambled or 14–3-3η-siRNA. **d** and **e** After Bel-7402 cells were transfected by scrambled or 14–3-3η-Flag, they were exposed to 0 or 500 μM hydrogen peroxide for 24 h. **b** and **d** The intracellular ROS levels; **c** and **e** Cell viability (determined in triplicate)

We previously found that in HCC cells, overexpression of 14–3-3η enhanced the cell viability and survival, leading to the resistance to sorafenib [12]. Here, in Bel/5-Fu cells, the expressions of 14–3-3η were significantly higher than those in parental Bel-7402 cells (Fig. 2a). So, we firstly validated the relationship between 14 and 3-3η and anti-oxidation. The 14–3-3η specific siRNA was transfected into Bel/5-Fu cells. As shown in Fig. 2b and c, knockdown of 14–3-3η further elevated the spontaneous intracellular ROS level and inhibited cell viability. In contrast, overexpression of 14–3-3η by transfecting the 14–3-3η plasmids into Bel-7402 cells significantly attenuated the hydrogen peroxide-induced ROS generation (Fig. 2d) and improved the cell survival (Fig. 2e). Similar results were also confirmed in another HCC cell line (Additional file 1: Figure S2).

**Fig. 3:**
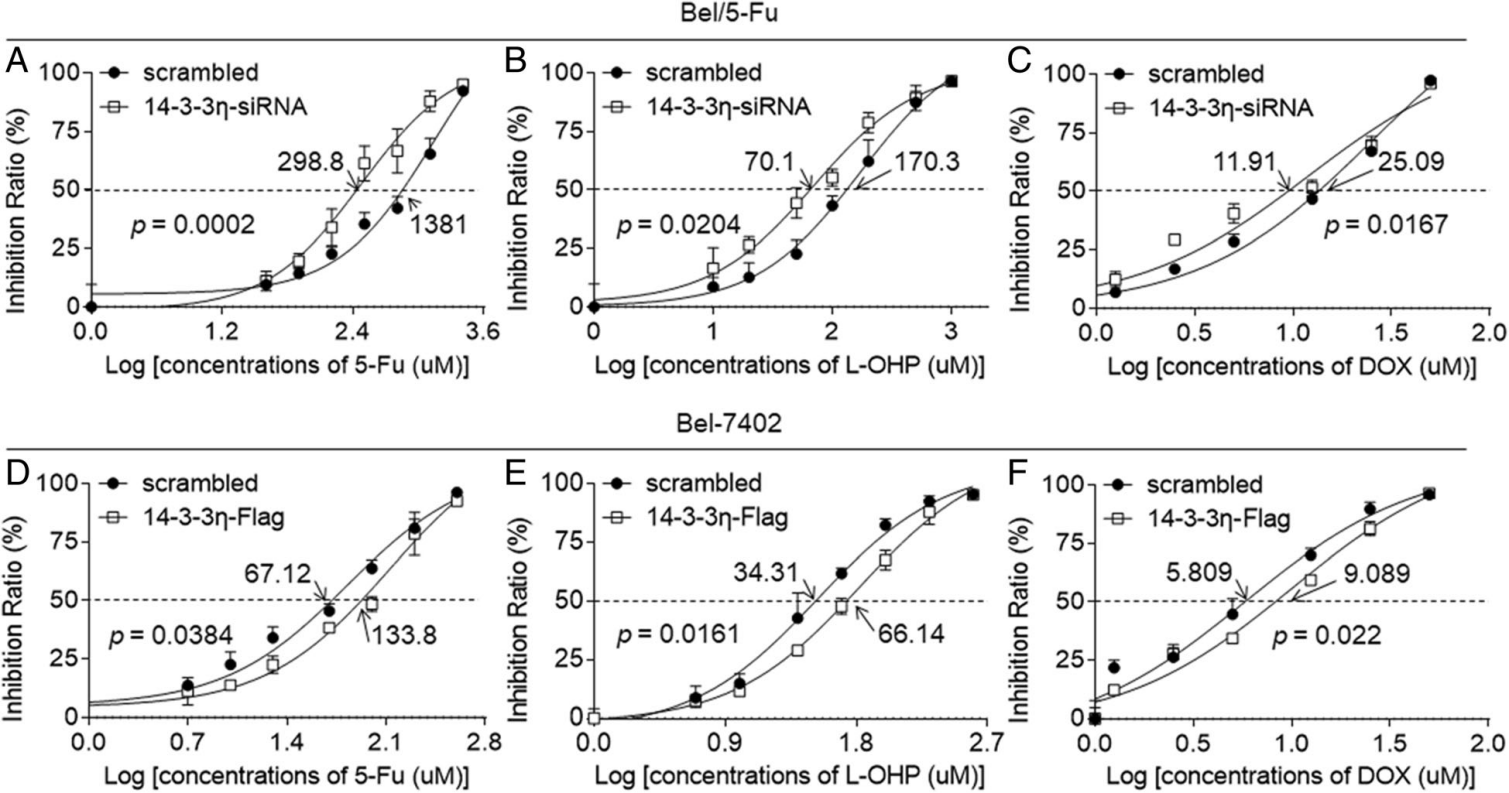
Effects of 14–3-3η on MDR in HCC cells: **a** to **c** Bel/5-Fu cells were transfected by scrambled or 14–3-3η-siRNA. **d** to **f** Bel-7402 cells were transfected by scrambled or 14–3-3η-Flag. Cells were treated by different concentrations of 5-fluorouracil, oxaliplatin, or doxorubicin for 24 h. The cell viability was determined in triplicate, and the IC_50s_ were calculated

Next we investigated if 14–3-3η could contribute the drug-resistance in Bel/5-Fu cells. As shown in Additional file 1: Figure S3, knockdown of 14–3-3η attenuated the expressions of *MDR1* and *MRP1* mRNAs. Meanwhile, the IC_50s_ (μM) of 5-fluorouracil, oxaliplatin, and doxorubicin for scramble- or 14–3-3η siRNA transfected Bel/5-Fu cells were: 1381 vs. 298.8, 170.3 vs. 70.1, and 25.09 vs. 11.91, respectively (Fig. 3a to c). In addition, we also determined if 14–3-3η could induce the MDR in parental Bel-7402 cells. As shown in Fig. 3d to f, the IC_50s_ (μM) of the above-mentioned three chemotherapeutic drugs for scramble- or 14–3-3η plasmids transfected Bel-7402 cells were 67.12 vs. 133.8, 34.31 vs. 66.14, and 5.809 vs. 9.089. Similar results were also confirmed in another HCC cell line (Additional file 1: Figure S4). Collectively, these results revealed that 14–3-3η was an effective anti-oxidant/ pro-survival factor, leading to the MDR of HCC cells.

### Potential mechanisms underlying 14–3-3η-induced/maintained MDR

**Fig. 4:**
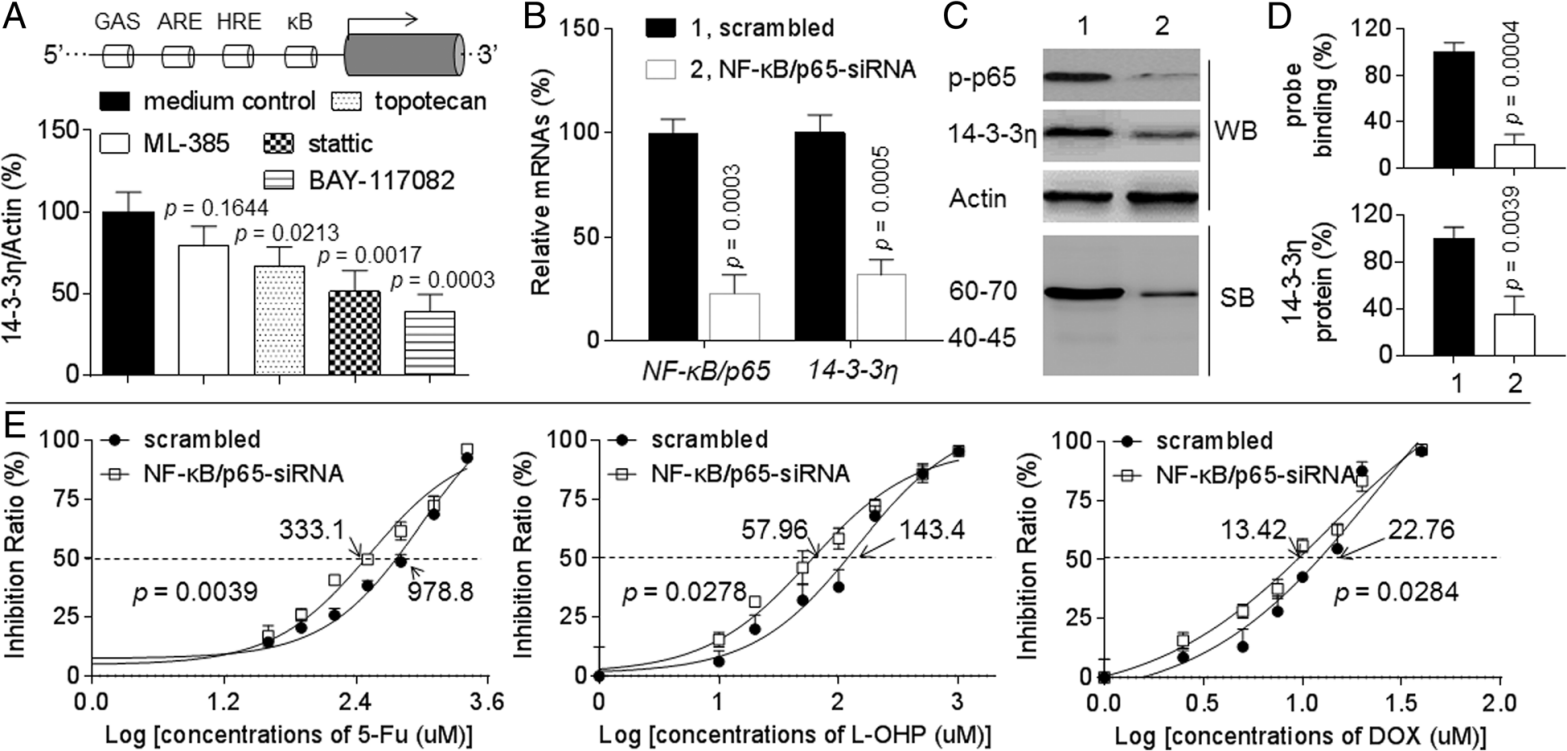
Effects of NF-κB on 14–3-3η and MDR in HCC cells: **a** Bel/5-Fu cells were treated by 20 μM of ML-385, topotecan, stattic, or BAY-117082 for 24 h. qRT-PCT analyses in triplicate of the *14–3-3η* mRNA. **b** to **e** Bel/5-Fu cells were transfected by scrambled or NF-κB/p65-siRNA. **b** qRT-PCT analyses in triplicate of the *NF-κB/p65* and *14–3-3η* mRNAs. **c**, top Western blot analyses of the expressions of p-p65 and 14–3-3η. **c**, bottom) Southwestern blot analyses of the binding of probe (containing the κB sequence located in 14–3-3η promoter) to NF-κB/p65. **d** Densitometric analysis. **e** The cell viability was determined in triplicate, and the IC_50s_ were calculated

Then, we focused on why the 14–3-3η was overexpressed in MDR-HCC cells. Via sequence alignment, we found four DNA binding elements located at the promoter region of *14–3-3η* gene. Those were the anti-oxidative response element (ARE, for NRF2), hypoxia response element (HRE, for HIF-1α), GAS (for STAT-3), and κB sequence (for NF-κB) (Fig. 4a). Interestingly, these four transcriptional factors play key roles in inducing/maintaining the anti-oxidation and MDR in HCC [22–24]. As shown in Fig. 4a, treatment of Bel/5-Fu cells with ML-385 (NRF2 inhibitor), topotecan (HIF-1α inhibitor), stattic (STAT-3 inhibitor), or BAY-117082 (NF-κB inhibitor) decreased the expression of *14–3-3η* mRNA from 100% (medium control group) to 79.38, 67.19, 51.57, and 38.27%, respectively.

The signal transductions among NRF2, HIF-1α, STAT-3, and NF-κB are complicated (involving both positive and negative regulations). Since the inhibition of NF-κB showed the best effect on 14–3-3η inactivation (Fig. 4a), we then determined the relationships between NF-κB and 14–3-3η. Here, knockdown of NF-κB in Bel/5-Fu cells attenuated the activation of NF-κB/p65 and 14–3-3η. Indeed it would inhibit the binding of probe (containing the κB sequence located in 14–3-3η promoter) to nuclear NF-κB/p65 (Fig. 4b to d), and decreased the IC_50s_ (μM) of 5-fluorouracil, oxaliplatin, and doxorubicin (978.8 vs. 333.1, 143.4 vs. 57.96, and 22.765 vs. 13.42, respectively, Fig. 4e). Meanwhile, we also confirmed that NF-κB was an up-stream regulator of 14–3-3η in other HCC cell lines (Additional file 1: Figure S5).

**Fig. 5:**
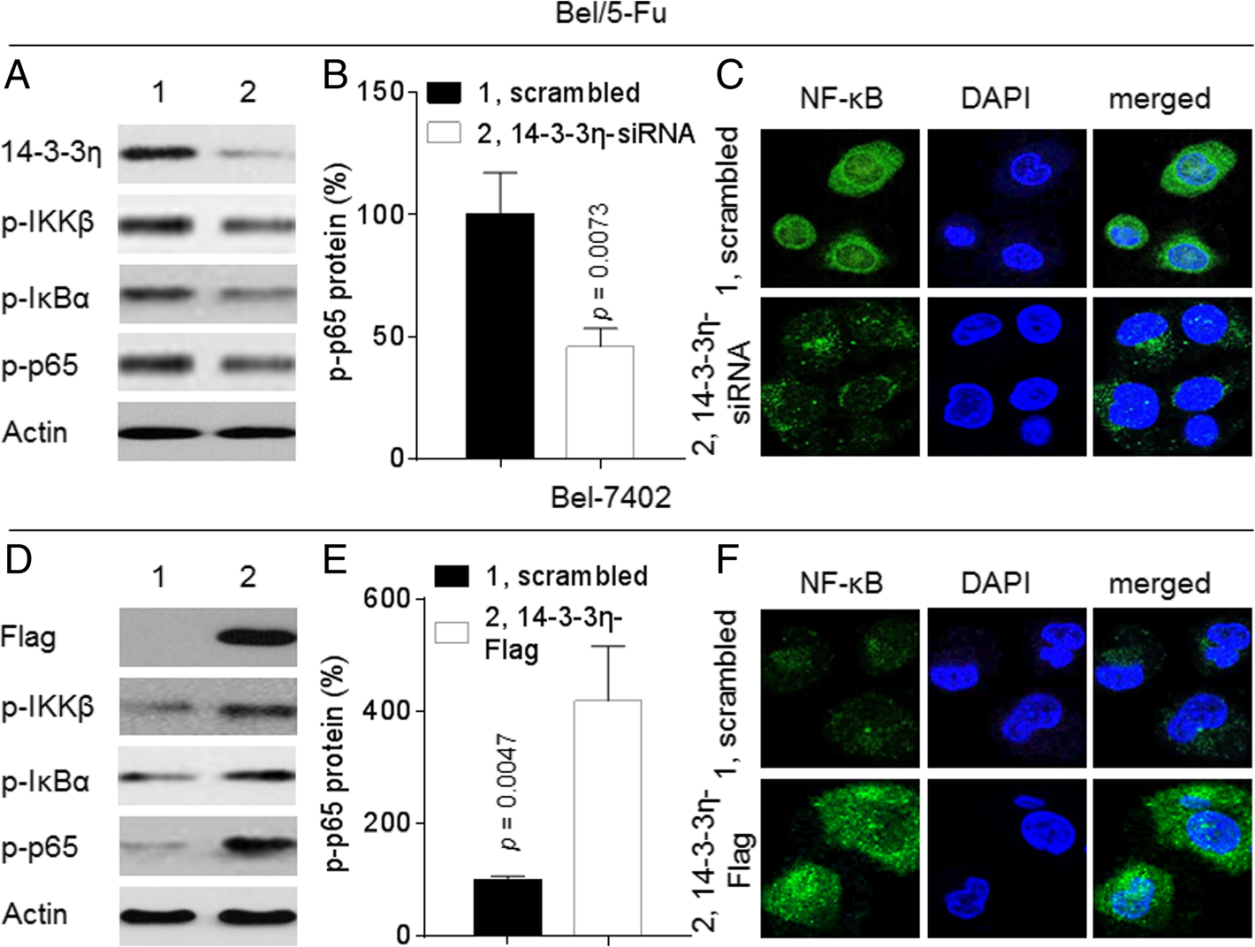
Effects of 14–3-3η on NF-κB in HCC cells: **a** to **c** Bel/5-Fu cells were transfected by scrambled or 14–3-3η-siRNA. **d** to **f** Bel-7402 cells were transfected by scrambled or 14–3-3η-Flag. **a** and **d** Western blot analyses of the expressions of p-IKKβ, p-IκBα, p-p65 and 14–3-3η/Flag. **b** and **e** Densitometric analysis. **c** and **f** Immunofluorescence analyses of the intracellular translocation of NF-κB/p65

Next, we further investigated whereby 14–3-3η induced/maintained MDR. Interestingly, studies revealed that some other 14–3-3 isoforms (such as ε and ζ) can also maintain the activity of NF-κB via enhancing the phosphorylation of NF-κB/p65 in HCC [25, 26]. In the present study, knockdown of 14–3-3η in Bel/5-Fu cells markedly suppressed the expression and/or nuclear translocation of p-IKKβ, p-IκBα, and p-NF-κB/p65 (Fig. 5a to c). On the contrary, overexpression of 14–3-3η in Bel-7402 cells showed the opposite effects (Fig. 5d to f). Similar results were also confirmed in other HCC cell lines (Additional file 1: Figure S6). Collectively, these results indicated that the 14–3-3η-mediated MDR in HCC cells was regulated by forming a positive feed-back loop with NF-κB.

### Identification of ATO as a characteristic 14–3-3η inhibitor in HCC cells

**Fig. 6:**
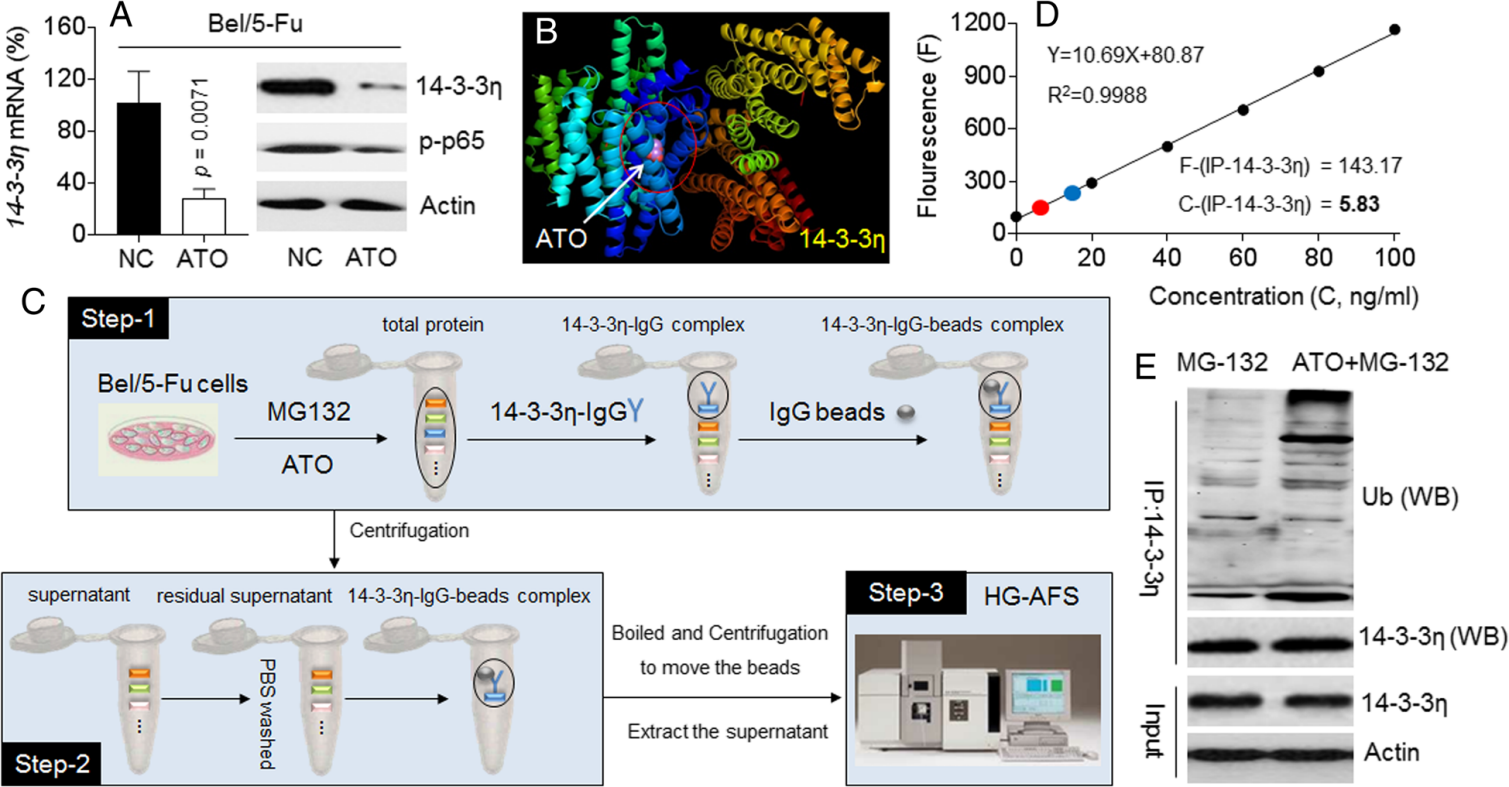
Relationships between ATO and 14–3-3η: **a** Bel/5-Fu cells were treated by 10 μM of ATO for 24 h. qRT-PCT analyses in triplicate (left) and Western blot (right) analyses of the expressions of 14–3-3η and/or p-p65. **b** PyMol software analyses of the binding of ATO to 14–3-3η. **c**The procedure of IP-AFS. **d** and **e** After Bel/5-Fu cells were pre-treated by 20 μM of MG-132 for 2 h, they were exposed to 10 μM of ATO for 6 h. The 14–3-3η was immunoprecipitated with the specific antibody. **d** AFS analyses of the concentration of ATO in 14–3-3η-immunoprecipitation complex. **e** Western blot analyses of the expression of ubiquitin in 14–3-3η-immunoprecipitation complex

Based on our previous findings, ATO is an effective NF-κB inhibitor in HCC cell line, MHCC97H [27, 28]. Here, as shown in Fig. 6a, ATO decreased the expression/ phosphorylation of NF-κB/p65 and 14–3-3η in Bel/5-Fu cells, suggesting that ATO might down-regulate 14–3-3η at the transcriptional level via the inhibition of NF-κB. Meanwhile, via a computer docking, we also found that ATO specifically bound to 14–3-3η protein, but not to other six 14–3-3 isoforms (Fig. 6b, and Additional file 1: Figure S7). Thus we adopted a novel approach by combining both IP and AFS techniques to reveal the interaction between 14 and 3-3η and ATO (Fig. 6c). The 14–3-3η was immunoprecipitated with its specific antibody and its ATO-binding status was determined by AFS to investigate if the immunoprecipitation complex contained ATO. As shown in Fig. 6d and Additional file 1: Figure S8, the concentration of ATO in positive control group (total protein) was 19.92 ng/ml. The concentration of ATO in 14–3-3η immunoprecipitated complex (experimental group) was 5.83 ng/ml. We almost did not detect the presence of ATO in negative control groups. Previous study revealed that ATO could target (bound to) and in turn induced the degradation of PML-RAR protein [29]. Here, we found that ATO treatment enhanced the ubiquitination of 14–3-3η in Bel/5-Fu cells (Fig. 6e). Collectively, these results indicated that, ATO inhibited the 14–3-3η at both transcriptional (indirectly, might via blocking NF-κB) and post-transcriptional (directly, might via targeting and inducing the ubiquitination and degradation of 14–3-3η) modifications in HCC cells.

### 14–3-3η was involved in ATO-reversed MDR

**Fig. 7:**
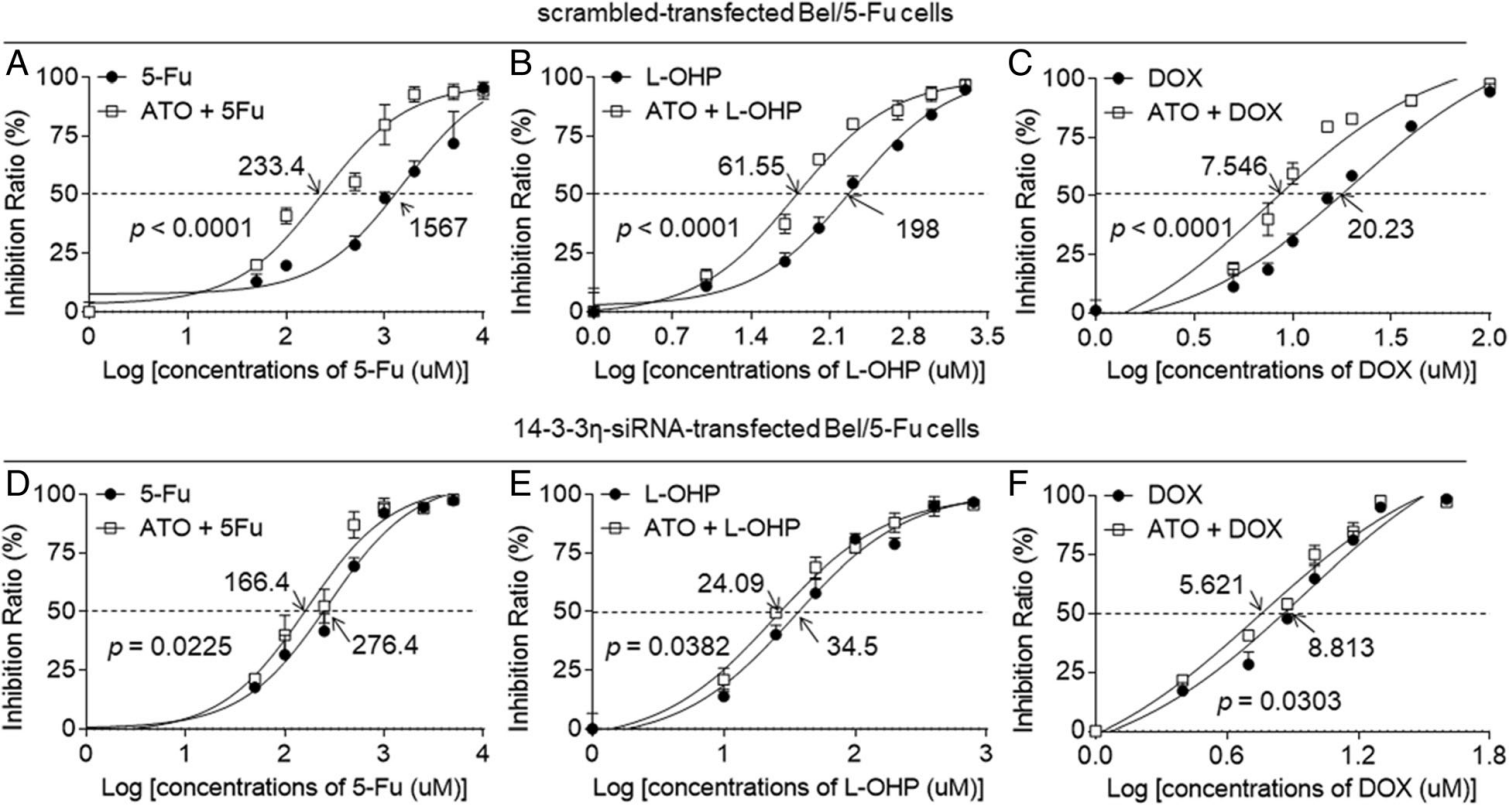
14–3-3η was involved in ATO-reversed MDR: The (**a** to **c**) scramble- or (**d** to **f**) 14–3-3η-siRNA transfected Bel/5-Fu cells were treated by different concentrations of 5-fluorouracil, oxaliplatin, and doxorubicin, in the absence of presence of 10 μM ATO for 24 h. The cell viability was determined in triplicate, and the IC50s were calculated

As shown in Additional file 1: Figure S9, the Bel/5-Fu cells were more sensitive to ATO in comparison with Bel-7402 cells [the IC50s (μM) were 17.69 vs. 32.46]. In addition, ATO-treatment decreased the expressions of *MDR1* and *MRP1* mRNAs (Additional file 1: Figure S10). Based on the above-mentioned results, we further suggested that, the relative higher level of 14–3-3η in Bel/5-Fu cells might be the mechanisms whereby they were more sensitive to ATO. For cancer chemotherapy, ATO was used with concentration of approximately 10.8 μM in the patients’ plasma [30]. Moreover, we previously revealed that 10 μM of ATO had no detectable cytotoxicity in normal hepatic or in non-drug resistant HCC cells [27, 28]. In the current study, the effects of 10 μM of ATO on the viabilities compared with non ATO-treated in Bel-7402 and Bel/5-Fu cells were 90.67 and 83.3%, respectively (Additional file 1: Figure S11). So we chose this concentration of ATO for further investigation. Here, the scramble-transfected Bel/5-Fu cells were treated by different concentrations of 5-fluorouracil, oxaliplatin, and doxorubicin, in the absence or presence of 10 μM ATO. The IC_50s_ (μM) of for chemotherapy alone group and chemotherapy combined with ATO treatment group were: 5-fluorouracil (1567 vs. 233.4), oxaliplatin (198 vs. 61.55), and doxorubicin (20.23 vs. 7.546, Fig. 7a to c). However, the IC_50s_ (μM) of chemotherapy alone group and chemotherapy combined with ATO treatment group were: 5-fluorouracil (276.4 vs. 166.4), oxaliplatin (34.5 vs. 24.09), and doxorubicin (8.813 vs. 5.621) in 14–3-3η-siRNA transfected Bel/5-Fu cells (Fig. 7d to f).

In scramble-transfected cells, co-treatment with ATO induced the reversal extents of drug resistance to 85.1% (5-fluorouracil), 68.9% (oxaliplatin), and 62.7% (doxorubicin). Nevertheless, in 14–3-3η-siRNA transfected cells, these reversal extents were further dropped to 39.79% (5-fluorouracil), 30.17% (oxaliplatin), and 36.2% (doxorubicin). Taken together, knockdown of 14–3-3η reduced the ATO-induced reversal extents of drug resistance, suggesting that 14–3-3η may be participated in the ATO-reversed MDR in HCC cells.

## Discussion

In current study, we used three first line chemotherapeutic agents, 5-fluorouracil, oxaliplatin, and doxorubicin. Classically, these three drugs inhibit the HCC progression through their respective pathways. 5-fluorouracil is a thymidylate synthase inhibitor and its metabolites can be incorporated into DNA, thus influence cell proliferation [31]. Oxaliplatin can covalently bind to guanine on the DNA chain and thus, block the replication and transcription of DNA [32]. Doxorubicin directly acts on DNA and inhibits DNA polymerase and thus can interfere both DNA and RNA synthesis [33]. Besides, 5-fluorouracil, oxaliplatin and doxorubicin are also reported to exhibit cytotoxic effect through increasing the ROS generation [34, 35]. Indeed, their effects on DNA damage and cell apoptosis can also be enhanced via oxidative stress. Furthermore, current study showed that Bel/5-Fu cell exhibited a relative higher level of ROS and possessed a capacity of anti-oxidation in comparison with Bel7402 cells. So, destroying the antioxidative capacity of drug-resistant cells could be expected to become a new approach to reverse the MDR.

NF-κB signaling pathway plays a key role in controlling the initiation and progression of human cancer [16, 20]. More recently, it has become clear that NF-κB signaling also has a critical role in chemotherapy resistance [36]. Here we found that, the elevated intracellular ROS level contributed to the activation of NF-κB in Bel/5-Fu cells (Additional file 1: Figure S12). NF-κB provides a mechanistic link between inflammation and cancer, and is a major factor controlling the ability of both pre-neoplastic and malignant cells to resist apoptosis-based tumor-surveillance mechanisms [37]. It might also regulate tumor angiogenesis and invasiveness, and the signaling pathways that mediate its activation, so as to become an attractive target for new chemo-preventive and chemo-therapeutic approaches [38].

The regulations of NF-κB by arsenic are very complex. In normal cells, the regulatory effect of arsenic on NF-κB depends on the dose of exposure. Generally, low dose of arsenic can activate NF-κB by inducing a moderate oxidative stress, while high dose of arsenic causes an excessive oxidative stress, leading to the inhibition of NF-κB [20, 21]. For cancer cells, our previous studies found that, via the demethylation-activation of microRNA-491, ATO effectively inhibited the activation of NF-κB in HCC [27, 28]. Our present study further revealed that, the targeted intervention of 14–3-3η also contributed to the ATO-induced inactivation of NF-κB. So, our results expand the understanding of the anti-cancer potential of ATO by identifying a novel mechanism whereby the ATO inhibited NF-κB.

ATO has been recognized as the first-line therapeutic agent for the treatment of acute promyelocytic leukemia [39]. In addition, ATO can suppress the progression in many solid tumors such as breast, colon, and liver [40–42]. We previous revealed that a relative lower concentration of ATO effectively attenuated migration/invasion, angiogenesis, and self-renewal in HCC cells [27, 28, 43]. Moreover, a recent clinical study revealed that there is a relatively prolonged overall/recurrence-free survival ratio in those patients treated with TACE plus an intravenous infusion of ATO in comparison with patients receiving TACE therapy alone [44]. Further, ATO can facilitate the anti-cancer activities of sorafenib in HCC cells [45]. In the rabbit HCC model, studies reveal that TACE used in combination with ATO had potent anticancer effects without significant hepatic or renal toxicities [46, 47]. Indeed, ATO has been approved for the treatment of human primary HCC by the State Food and Drug Administration of China [48, 49]. In addition, unlike other chemotherapy drugs, ATO has been recognized as a molecular targeted drug in the “Standard for Diagnosis and Treatment of Primary HCC in China (2017)”, as it can target and in turn induce the degradation of PML-RAR protein [29]. Here, we found that 14–3-3η played an important role in inducing/maintaining the antioxidation/MDR state. Nevertheless, there is no identified inhibitor for 14–3-3η up to date, so our present study suggested for the first time that ATO is an effective chemical inhibitor for 14–3-3η.

## Conclusion

**Fig. 8:**
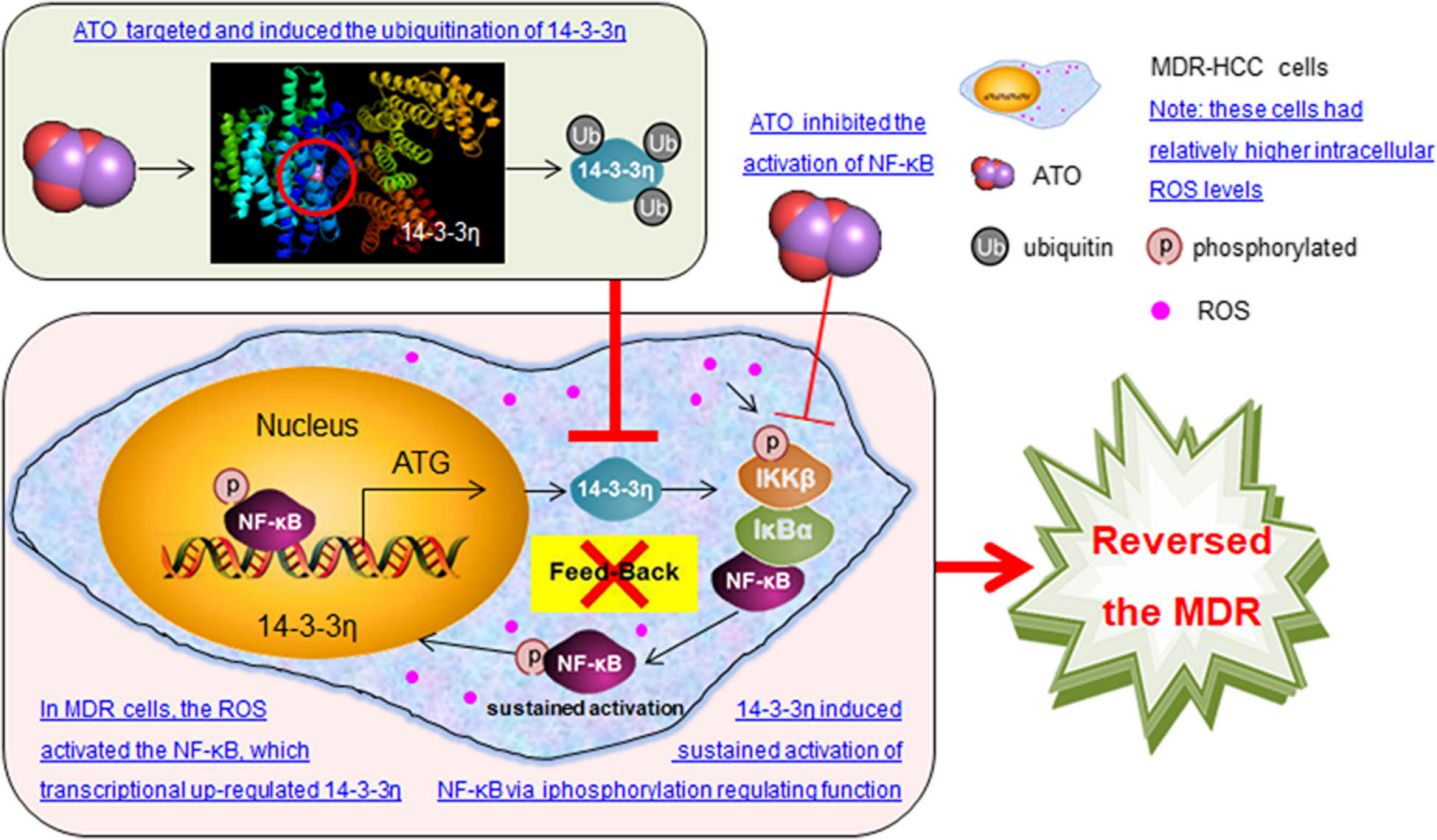
Arsenic Trioxide Reverses the Chemoresistance in Hepatocellular Carcinoma: A Targeted Intervention of 14–3-3η/NF-κB Feedback Loop

In conclusion, Bel/5-Fu cells exhibited a relative higher level of ROS, which activated NF-κB, leading to the transcriptionally up-regulation of 14–3-3η. NF-κB/14–3-3η can form a positive feed-back loop to induce/maintain the MDR phenotype in HCC cells. ATO could abolish this feed-back loop by either blocking of NF-κB signaling and 14–3-3η transcription, or by targeting 14–3-3η for its ubiquitination and degradation. These processes abolished the antioxidation mechanisms employed by Bel/5-Fu cells and reversed the MDR (Fig. 8).

## Additional file

Additional file 1: Arsenic Trioxide Reverses the Chemoresistance in Hepatocellular Carcinoma: A Targeted Intervention of 14–3-3η/NF-κB Feedback Loop. (PDF 397 kb)

## Abbreviations

ATO: Arsenic trioxide
HCC: Hepatocellular carcinoma
MDR: Multi-drug resistance
NF-κB: Nuclear factor kappa B
ROS: Reactive oxygen species
