# Supplementary material for: Arsenic trioxide reverses the chemoresistance in hepatocellular carcinoma: a targeted intervention of 14–3-3η/NF-κB feedback loop
Source: J Exp Clin Cancer Res. 2018 Dec 20;37:321. doi: 10.1186/s13046-018-1005-y (PMC6302299; doi:10.1186/s13046-018-1005-y)
Supplement: Supplementary file 1 — Arsenic Trioxide Reverses the Chemoresistance in Hepatocellular Carcinoma: A Targeted Intervention of 14–3-3η/NF-κB Feedback Loop. (PDF 397 kb) [file 13046_2018_1005_MOESM1_ESM.pdf]

Arsenic Trioxide Reverses the Chemoresistance in Hepatocellular Carcinoma: A Targeted Intervention of 14-3-3η/NF-κB Feedback Loop

Yongxin Qiu, Yi Dai, Chi Zhang, Ye Yang, Ming Jin, Wenqi Shan, Jian Shen, Ming Lu, Zhaoyang Tang, Liang Ju, Yuting Wang, Ruonan Jiao, Yunwei Xia, Guangming Huang, Lihua Yang, Yuan Li, Jianping Zhang, Vincent Kam Wai Wong, Zhihong Jiang

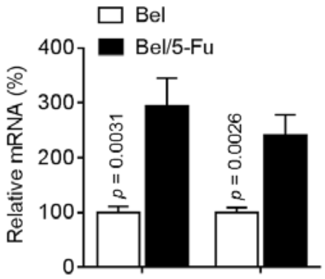

Supplement Fig. 1: The primers for *MDR1* were F: 5'-gctcatcgtttgtctacagtt-3', and R: 5'-acaatgactccatcatcgaa -3', while the primers for *MPR1* were 5'-ggatcatgctcatttctg-3', and R: 5'-aagtgatgtcacgaaacaggt-3'; qRT-PCT analyses in triplicate of the *MDR1* (left) and *MRP1* (right) mRNAs.

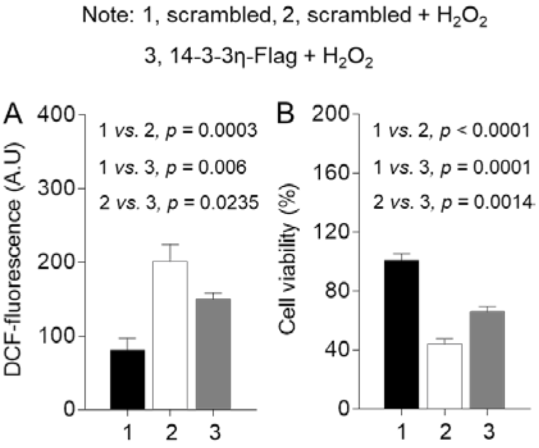

Supplement Fig. 2: After HepG2 cells were transfected by scrambled or 14-3-3η, they were exposed to 0 or 500 μM hydrogen peroxide for 24 h. (A) The intracellular ROS levels (determined in triplicate). (B) Cell viability (determined in triplicate).

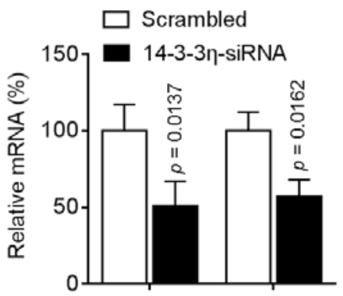

Supplement Fig. 3: Bel/5-Fu cells were transfected by scrambled or 14-3-3η-siRNA, qRT-PCT analyses in triplicate of the *MDR1* (left) and *MRP1* (right) mRNAs.

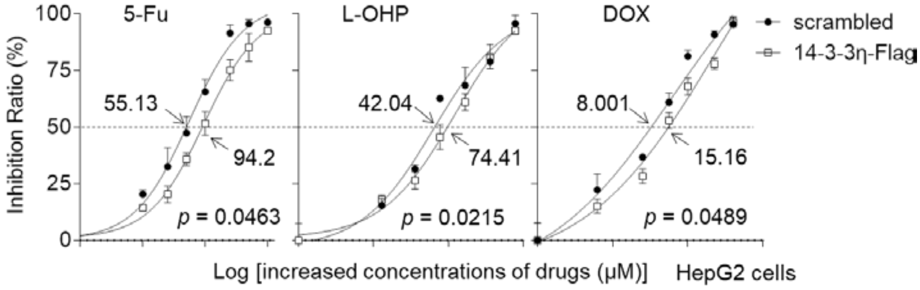

Supplement Fig. 4: HepG2 cells were transfected by scrambled or 14-3-3η. Cells were treated by different concentrations of 5-fluorouracil, oxaliplatin, or doxorubicin for 24 h. The cell viability was determined in triplicate, and the IC_{50s} were calculated.

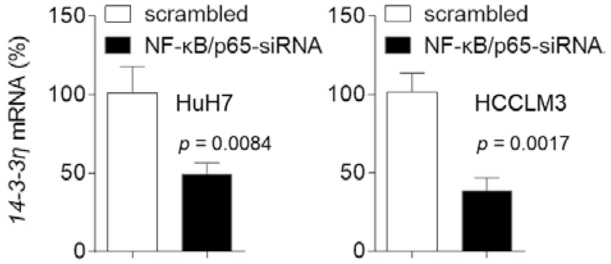

Supplement Fig. 5: HuH7 (left) or HCCLM3 (right) cells were transfected by scrambled or p65-siRNA. qRT-PCT analyses in triplicate of the *14-3-3η* mRNA. We chose these two cells for investigating the effects of NF-κB on 14-3-3η because they have relative higher background phosphorylation levels of NF-κB/p65 [3, 4].

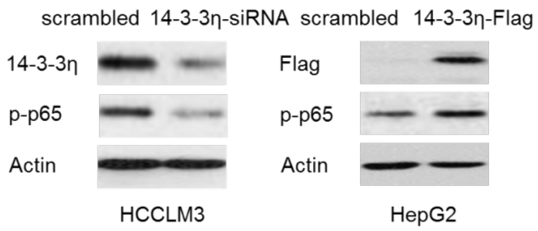

Supplement Fig. 6: HCCLM3 cells were transfected by scrambled or 14-3-3η-siRNA, while HepG2 cells were transfected by scrambled or 14-3-3η. Western blot analyses of the expressions of p-p65 and 14-3-3η/Flag.

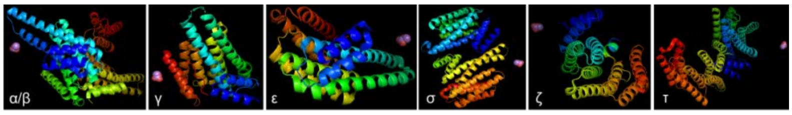

Supplement Fig. 7: PyMol software analyses the binding of ATO to 14-3-3 isoforms.

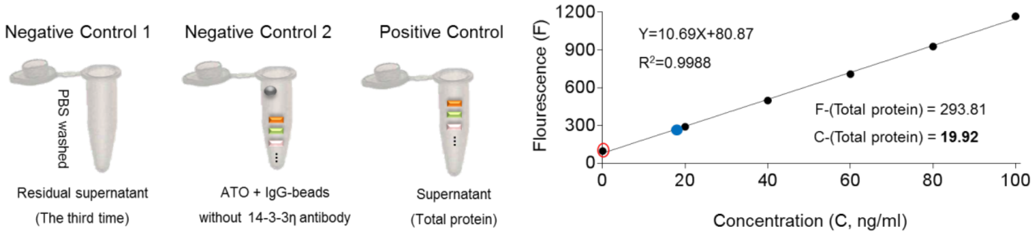

Supplement Fig. 8: AFS analyses of the concentration of ATO in negative or positive groups. The concentration of ATO in positive control group (total protein) was 19.92 ng/ml (The blue dot). We almost did not detect the presence of ATO in negative control groups (The ranges of flourescences were showed as the red circle).

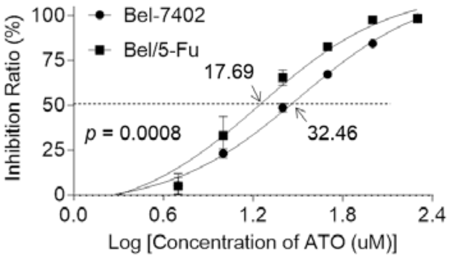

Supplement Fig. 9: Bel-7402 or Bel/5-Fu cells were treated by different concentrations of ATO for 24 h. The cell viability was determined in triplicate, and the IC_{50s} were calculated.

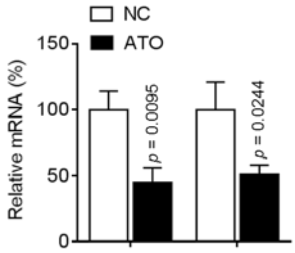

Supplement Fig. 10: Bel/5-Fu cells were treated by 10 μM of ATO for 24 h, qRT-PCT analyses in triplicate of the *MDR1* (left) and *MRP1* (right) mRNAs.

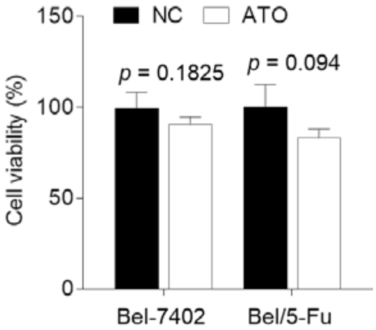

Supplement Fig. 11: Bel-7402 or Bel/5-Fu cells were treated by 10 μM of ATO for 24 h. The cell viability was determined in triplicate.

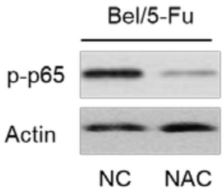

Supplement Fig. 12: Bel/5-Fu cells were treated by 10 μM of NAC for 6 h. Western blot analyses of the phosphorylation of NF-κB/p65.

References

1. Fu X, et al. Exosomal microRNA-32-5p induces multidrug resistance in hepatocellular carcinoma via the PI3K/Akt pathway. J Exp Clin Cancer Res. 2018; 37(1): 52.
2. Shen J, et al. 14-3-3eta is a novel growth-promoting and angiogenic factor in hepatocellular carcinoma. J Hepatol. 2016; 65(5): 953-962.

3. Jiang F, et al. Inhibition of TGF-beta/SMAD3/NF-kappaB signaling by microRNA-491 is involved in arsenic trioxide-induced anti-angiogenesis in hepatocellular carcinoma cells. Toxicol Lett. 2014; 231(1): 55-61.
4. Wang X, et al. Arsenic trioxide attenuates the invasion potential of human liver cancer cells through the demethylation-activated microRNA-491. Toxicol Lett. 2014; 227(2): 75-83.
